# Aberrant Pancreatic Tissue in a Mediastinal Enteric Duplication Cyst: A Rarity with Review of Literature

**DOI:** 10.1155/2017/7294896

**Published:** 2017-04-26

**Authors:** Meha Mansi, Nidhi Mahajan, Sonam Mahana, C. R. Gupta, Anup Mohta

**Affiliations:** ^1^Department of Pathology, Chacha Nehru Bal Chikitsalaya, New Delhi, India; ^2^Department of Pediatric Surgery, Chacha Nehru Bal Chikitsalaya, New Delhi, India

## Abstract

Mediastinal enteric duplication cysts are a rare congenital malformation encountered mainly in neonates and infants. It is a distinct entity within the family of foregut duplication cysts. It can present with respiratory distress due to mass effect and hence surgical excision is the preferred treatment. Histologically, it is characterised by a double layered smooth muscle wall with intestinal lining epithelium. We report a case of mediastinal enteric duplication cyst with aberrant pancreatic tissue in a neonate due to its rarity and early presentation. A neonate presented with respiratory distress and a cystic mass in the right posterior mediastinum. The lesion was excised and on histopathological analysis the diagnosis of mediastinal enteric duplication cyst was made. Also, aberrant pancreatic tissue which has been reported rarely was noted in this case. We discuss this case and review similar cases reported in literature.

## 1. Introduction

Mediastinal enteric duplication cysts are rare congenital malformations seen in neonates and infants. They are a type of foregut duplication cysts, with the other subtypes being bronchogenic and esophageal cyst [1]. The clinical presentation of these cysts depends upon the anatomic site of the gut involved, mass effect of the cyst, and symptoms or complications related to the ectopic mucosal lining which may be present in the cyst. This being extremely important in children as functional gastric mucosa may lead to excessive acid secretion and perforation and ectopic pancreatic tissue may lead to hypoglycaemic attacks. Both the presentations are difficult to diagnose especially in paediatric population. We report a case of mediastinal enteric duplication cyst with aberrant pancreatic tissue in the submucosa for its rarity and dubious radiological features and also review in parallel the literature.

## 2. Case Report

**Figure 1:**
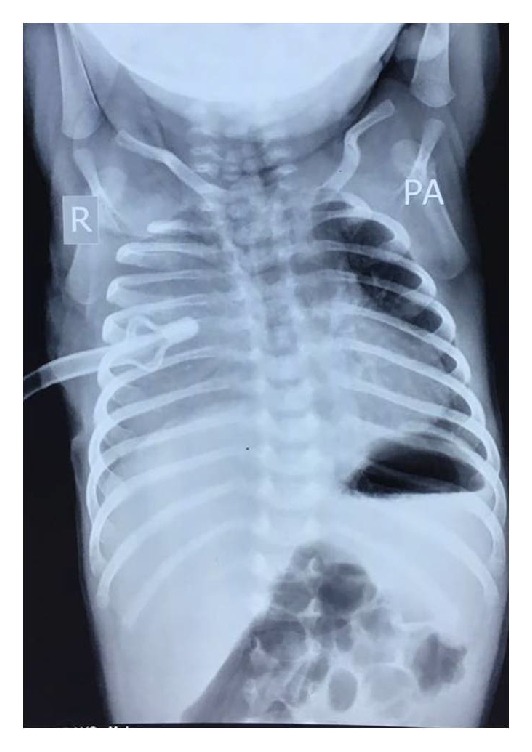
Chest radiograph showing a homogeneous opacity in the right middle and lower lung fields with shift of mediastinum to left.

**Figure 2:**
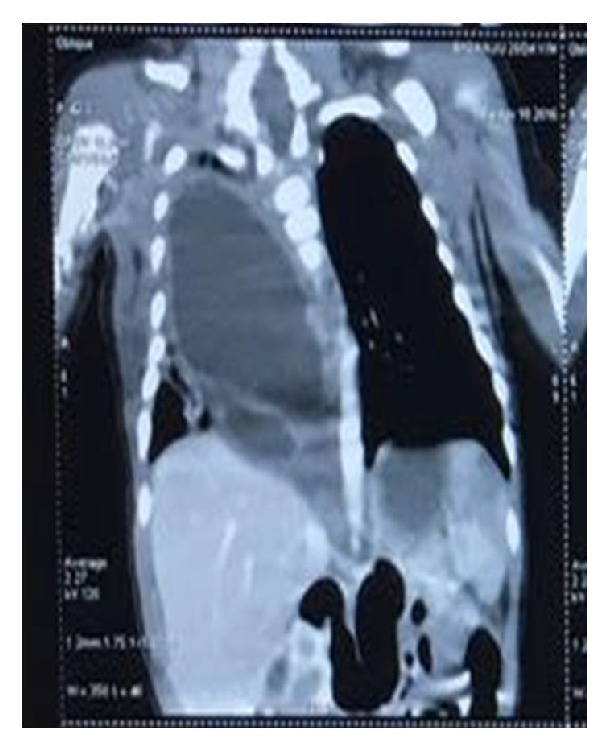
Computed tomography scan showed a well-defined fluid attenuation lesion with broad base towards the mediastinum with few loculations.

A 29-day-old neonate weighing 4.2 kg was referred with respiratory distress since day four of life. The child was born of a nonconsanguineous marriage, at 37 weeks, with birth weight of 3.5 kg. The pregnancy and delivery were unremarkable. At admission, the child had poor activity with respiratory rate of 30/minute and pulse rate of 140/minute. Heart sounds were normal. There was decreased air entry on the right side. Biochemical investigations were normal. On the X-ray, a homogeneous opacity was seen in the right middle and lower lung fields with shift of mediastinum to the left (Figure 1). No vertebral defect was seen. In view of pneumothorax, a chest tube was put. Ultrasound, however, showed a well-defined cystic lesion in the right lower lung field. Contrast-enhanced computerised tomography confirmed the presence of a well-defined multilocular cystic collection in the posterior aspect of middle and lower zone of right lung (Figure 2). The hematological profile and serum biochemistry were within the normal limits. Based on the clinical and radiological findings, differential diagnosis of a duplication cyst or congenital cystic adenomatoid malformation was suggested. The patient underwent right thoracotomy with excision of the cyst. The chest was approached by right thoracotomy through the 5th intercostal space. Intraoperatively, a large tense cyst measuring 5 × 4 × 3.5 cms was noted arising from the posterior mediastinum densely adherent to the lower esophagus and the diaphragm. No communication with the esophageal lumen was present. The cyst was slowly dissected free from both the structures. The cyst could be separated easily from the lung parenchyma. The vertebra was also normal.

**Figure 3:**
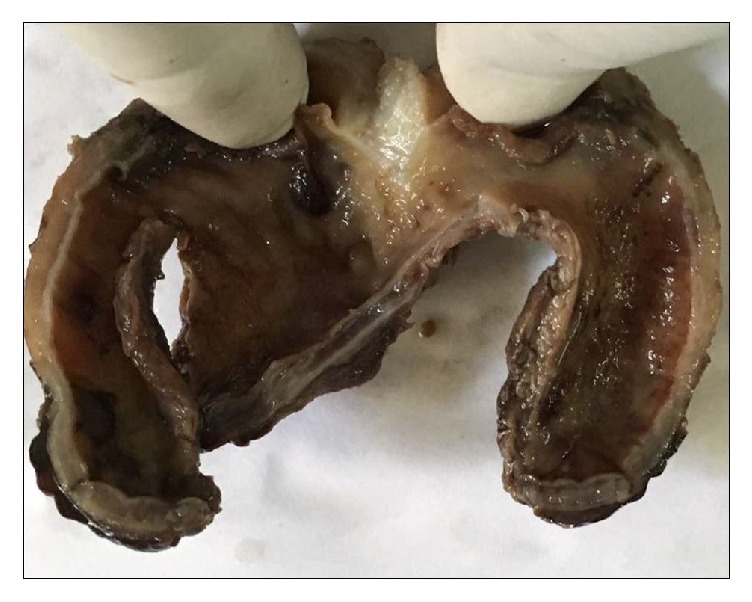
Specimen comprising of a unilocular mass with thickened wall and inner surface markedly congested.

**Figure 4:**
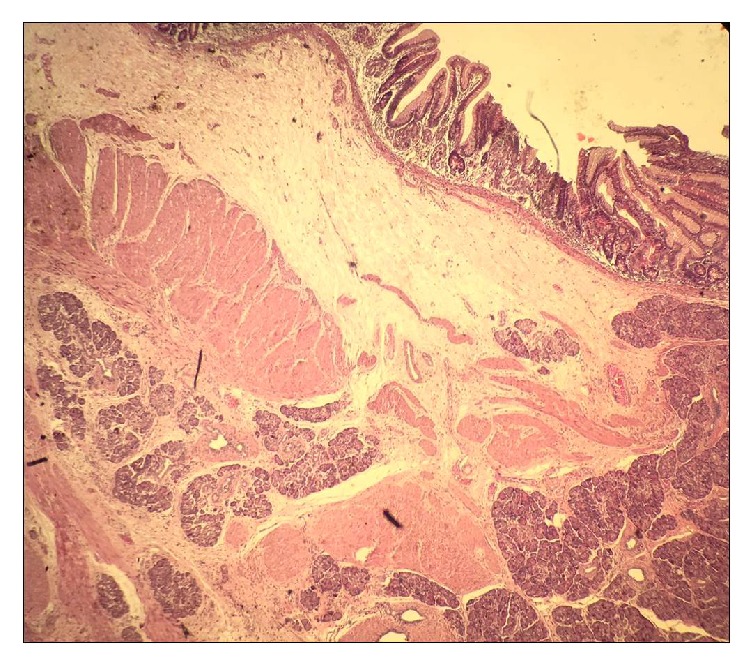
Microphotograph showing scanner view of the cyst wall showing double layered intestinal wall with normal intestinal mucosa and pancreatic rests within the muscularis (Hematoxylin & Eosin, 40x).

**Figure 5:**
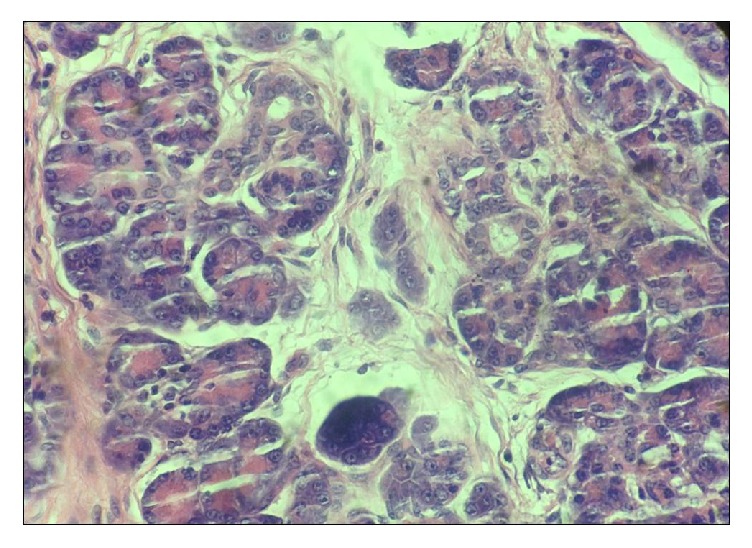
Microphotograph showing acini with cells having abundant eosinophilic granular cytoplasm (Hematoxylin & Eosin, 400x).

On gross pathological examination, the cyst was greyish brown, multiloculated with wall thickness of 0.5 cm (Figure 3). Microscopic sections showed a cyst wall lined by intestinal epithelium with a double layered muscular wall. Aberrant pancreatic tissue was noted in the submucosa and muscularis propria (Figures 4 and 5). Though the Islets of Langerhans were not seen, isolated endocrine cells were seen scattered amidst the exocrine acini. Final histopathological diagnosis of an enteric duplication cyst with ectopic pancreatic rest was suggested. The postoperative course was uneventful. The patient is doing well at follow-up and has not developed any complications.

## 3. Discussion

The term duplication cyst was first introduced in 1711 by Blassius and Bremer [1]. Gastrointestinal tract duplication cysts are rare congenital malformations seen in infants and children. Midgut duplication cysts are the commonest, followed by foregut and hindgut duplication cysts. Foregut duplication cysts constitute 10% of all mediastinal tumors [1]. They are further classified on the basis of their embryonic origin into bronchogenic, esophageal, and enteric duplication cysts.

Mediastinal enteric cyst is rare and in 60% cases these cysts are diagnosed in neonates and infants with a slight male preponderance [2]. In a case series reported by Cohen et al., out of 15 foregut cysts, none was of enteric type [1]. Enteric cysts associated with vertebral anomalies are called neurenteric cysts. These are usually seen associated with vertebral anomalies like vertebral fusion, scoliosis, anterior and posterior spina bifida, hemivertebrae, diastomyelia, and absence of vertebra [3].

Mediastinal enteric cyst is usually seen in the right posterior mediastinum. It normally presents with pressure symptoms like respiratory distress due to pressure on the bronchi or lung, cough, cyanosis, retrosternal pain, and dysphagia. Children may present with recurrent chest infections. Hematemesis may occur if there is a communication with the esophagus. Ectopic gastric mucosa can be present, leading to peptic ulceration, haemorrhage, or perforation. The current case also presented with respiratory distress and showed a left mediastinal shift.

X-ray of a duplication cyst is an extremely important first-line investigation. Lateral and posterior-anterior views detect maximum lesions and show a homogeneous mass in the posterior mediastinum, usually on the right side. Ultrasonography has limited value; however, on endoscopic ultrasound, a duplication cyst appears as an anechoic or hypoechoic homogeneous cystic lesion with regular margins. Contrast-enhanced computerised tomography confirmed the presence of a well-defined homogeneously enhancing multilocular cystic lesion located in or adjacent to the wall of the part of alimentary canal.

Histopathologically, the cyst shows smooth muscle wall lined by enteric type epithelium. Ectopic gastric lining may be present. Pancreatic tissue in the mediastinum appears as a part of germ cell tumors or as pseudocysts; its presence in the wall of enteric cysts is extremely rare and to the best of our knowledge has been reported only thrice in literature [5] (Table 1). The etiology behind an ectopic rest in the present case is unclear but it has been postulated as pancreatic cell migration and heteroplasia [6]. It is of importance as functional pancreatic tissue may produce insulin and lead to erratic unexplained hypoglycemic episodes in such patients. In the current case, the cyst wall showed unremarkable intestinal lining mucosa with a good volume of ectopic pancreatic rest which was nonfunctional in our case as child had no history suggestive of hypoglycemia and normal biochemistry.

Differentiation from other cystic lesions of the mediastinum like neurenteric cyst, pericardial cyst, thymic cyst, bronchogenic cysts, meningocele, lymphangioma, mature cystic teratoma, cystic schwannomas, cystic thymomas, and cystic tubercular lymphadenitis is important as it has important implications in further management of the patient.

Neurenteric cyst shows variable lining epithelium from respiratory, transitional to squamous. The wall shows variably thickened muscularis layer along with few nerve twigs and dystrophic neurons. There may be evidence of calcification. Pericardial cysts are usually thin walled, benign, filled with clear fluid, and lined by mesothelium. Thymic cysts may be unilocular or multilocular and are thin walled tense cysts filled with brown fluid showing pericystic fibrosis with inflammation, haemorrhage, and cholesterol clefts in the wall [7]. Lymphangiomas are extremely rare, usually present later in life till they obtain significant sizes, and histologically characterised by flattened endothelial lining of the wall filled with lymphatic fluid. Cystic teratomas are rare and usually seen in young adults and comprise of elements from all germ cell lines, especially hair follicles and sebaceous glands.

Thoracoscopic surgical excision of the cyst is the mainstay of treatment with comprehensive supportive care. This resolves the majority of the cases and is associated with minimal morbidity. Few cases which have esophageal or vertebral connections need thoracotomy. In our case, the cyst was quite large in size and expertise in thoracoscopy was not available; hence, the patient was taken up for thoracotomy. To conclude, early intervention is needed in these cases before the patient becomes symptomatic as these lesions, once symptomatic, tend to be associated with higher intraoperative complications and, if left untreated, may be complicated by perforation, obstruction, or haemorrhage. It is important for the pathologists to be aware of the vast differential diagnosis of mediastinal cysts on histopathology which would require thorough sampling to arrive at a correct diagnosis. An active search for ectopic gastric/pancreatic rests is recommended, for both its academic importance and clinical correlation in symptomatic patients.

## Figures and Tables

**Table 1:** 

Study	Age and sex	Duplication cyst type	Ectopic tissue type
Qazi et al., 1990 [4]	8 years/M	Esophageal gastroenteric duplication cyst	Pancreatic
Prasad et al., 2002 [5]	1 day/M	Mediastinal enteric duplication cyst	Pancreatic
Anagnostou et al., 2009 [2]	2 days/F	Mediastinal enteric duplication cyst	Pancreatic
Present case	4 days/M	Mediastinal enteric duplication cyst	Pancreatic
